# A Good Preparation

**Published:** 1876-08

**Authors:** 


*A Good Preparation.*—We have used a preparation of cod-liver oil chemically combined with soluble phosphate of lime, with very great advantage in those cases calling for cod-liver oil. The union of cod-liver oil and phosphate of lime is one much used and recommended by physicians, and the preparation referred to above will give the best possible form for the administration of these. The preparation employed was that of **A.** Jas., druggist and chemist, No. 103 Chartres street, New Orleans. It has the endorsement of such medical men as Prof. Joseph Jones, and will be much prized by the profession when fully known. G.

